# A method for accurate detection of genomic microdeletions using real-time quantitative PCR

**DOI:** 10.1186/1471-2164-6-180

**Published:** 2005-12-13

**Authors:** Rosanna Weksberg, Simon Hughes, Laura Moldovan, Anne S Bassett, Eva WC Chow, Jeremy A Squire

**Affiliations:** 1 Program in Genetics and Genomic Biology, The Research Institute, The Hospital for Sick Children, Toronto, Canada; 2 Division of Clinical and Metabolic Genetics, Department of Paediatrics, The Hospital for Sick Children, Toronto, Canada; 3 Queen Mary's School of Medicine and Dentistry, London, UK; 4 Clinical Genetics Research Program, Centre for Addiction and Mental Health, and Department of Psychiatry, University of Toronto, Toronto, Canada; 5 Ontario Cancer Institute and Department of Laboratory Medicine, Pathology and Medical Biophysics, University of Toronto, Toronto, Canada

## Abstract

**Background:**

Quantitative Polymerase Chain Reaction (qPCR) is a well-established method for quantifying levels of gene expression, but has not been routinely applied to the detection of constitutional copy number alterations of human genomic DNA. Microdeletions or microduplications of the human genome are associated with a variety of genetic disorders. Although, clinical laboratories routinely use fluorescence *in situ *hybridization (FISH) to identify such cryptic genomic alterations, there remains a significant number of individuals in which constitutional genomic imbalance is suspected, based on clinical parameters, but cannot be readily detected using current cytogenetic techniques.

**Results:**

In this study, a novel application for real-time qPCR is presented that can be used to reproducibly detect chromosomal microdeletions and microduplications. This approach was applied to DNA from a series of patient samples and controls to validate genomic copy number alteration at cytoband 22q11. The study group comprised 12 patients with clinical symptoms of chromosome 22q11 deletion syndrome (22q11DS), 1 patient trisomic for 22q11 and 4 normal controls. 6 of the patients (group 1) had known hemizygous deletions, as detected by standard diagnostic FISH, whilst the remaining 6 patients (group 2) were classified as 22q11DS negative using the clinical FISH assay. Screening of the patients and controls with a set of 10 real time qPCR primers, spanning the 22q11.2-deleted region and flanking sequence, confirmed the FISH assay results for all patients with 100% concordance. Moreover, this qPCR enabled a refinement of the region of deletion at 22q11. Analysis of DNA from chromosome 22 trisomic sample demonstrated genomic duplication within 22q11.

**Conclusion:**

In this paper we present a qPCR approach for the detection of chromosomal microdeletions and microduplications. The strategic use of *in silico *modelling for qPCR primer design to avoid regions of repetitive DNA, whilst providing a level of genomic resolution greater than standard cytogenetic assays. The implementation of qPCR detection in clinical laboratories will address the need to replace complex, expensive and time consuming FISH screening to detect genomic microdeletions or duplications of clinical importance.

## Background

Array comparative genomic [1-3] and FISH-based methods [4] have been widely used for the detection of DNA copy number changes. However, the resolution of commercially available DNA arrays can be too low to detect microdeletions or microduplications [5,6], whilst FISH is generally only useful when the regions of interest have been previously defined. Currently, DNA arrays providing full coverage of the human genome are not widely available and too expensive to diagnostically screen large numbers of patients. Moreover, the findings that are emerging from recent array comparative genomic hybridization studies indicate that significant validations of both controls and patient populations will be required to make phenotype-genotype interpretations in a clinical context. Similarly, standard FISH methods are time consuming, costly and suffer the significant limitation that some patients with uniquely localized microdeletions or duplications may yield normal clinical FISH findings because the probe set used does not map precisely to the entire region of deletions/duplication.

In this study we have chosen to validate the use of qPCR technology for detection of microdeletions or microduplications using Velocardiofacial Syndrome (VCFS) or chromosome 22q11 deletion syndrome (22q11DS) as a test model. The frequency of the causative deletion for 22q11DS in the general population is 1 in 3000 live births, making it one of the most common constitutional genomic alterations found in humans [7]. 22q11DS is suspected in individuals with characteristic clinical findings and is confirmed in most cases by detection of a sub-microscopic deletion using FISH.

The currently accepted clinical laboratory assay for 22q11DS uses the TUPLE1 FISH probe, which is located within a typically deleted region of approximately 3 Mb. Although this assay can detect the majority of affected patients (85–90%), many patients with phenotypic features of 22q11DS have no deletion detectable by FISH testing. As a consequence these patients will go undiagnosed due to the presence of atypical deletions that map outside the area covered by the TUPLE1 probe [8-12]. In addition, there have been reports of individuals with some features of 22q11DS with microduplications of 22q11.2 [13]. Unfortunately, clinical FISH assays are not usually capable of detecting such duplications, so alternative methods, such as FISH analysis of interphase nuclei, are required [14]. However, such techniques require advanced optical instrumentation, presently only used in specialized research laboratories. Alternative molecular technologies that could potentially be used to screen deletions in the diagnose of 22q11DS is multiplex ligation-dependent probe amplification (MLPA) [15] or microsatellites marker analysis [10].

The novel approach validated in this study utilized qPCR rather than FISH to detect copy number alterations (microdeletions and microduplications) in patient DNA. This approach had several advantages. Primers were selected within regions of unique sequence utilizing publicly available sequence databases [16-24]. This method can allow for the production of a high-resolution map of any region of interest; the 22q11DS region is used in this example. The qPCR technique provides a quantitative measurement of DNA copy number and accurately characterizing chromosomal breakpoints. This method will therefore permit the identification of individuals who would otherwise go undetected by the currently available clinical FISH methods. In addition, qPCR provides greater flexibility and adaptability, whilst being less technically challenging than FISH, thus making it more appropriate for use in a large number of laboratories. Furthermore, the qPCR technique only takes a fraction of the time usually required for FISH, which allows for multiple samples and multiple primer sets to be studied in parallel, using convenient and cost effective high throughput analysis. The method described in this paper has been evaluated using patient and controls samples with known copy number changes on 22q11. The approach can be readily adapted for molecular diagnostics of any region of the genome suffering recurrent constitutional genomic deletion or duplication.

## Results

### Fish results

The twelve patients utilized in this study have previously been analysed in a clinical laboratory by FISH using the TUPLE 1 probe. Using this test, six of the patients (group 1) were identified as 22q11 deletion positive whilst the other six (group 2) and the four controls showed normal cytogenetic results. The DNA sample known to have three copies of 22q11 (trisomy for chromosome 22) had been previously analysed by FISH [25].

### Primer design

**Figure 1:**
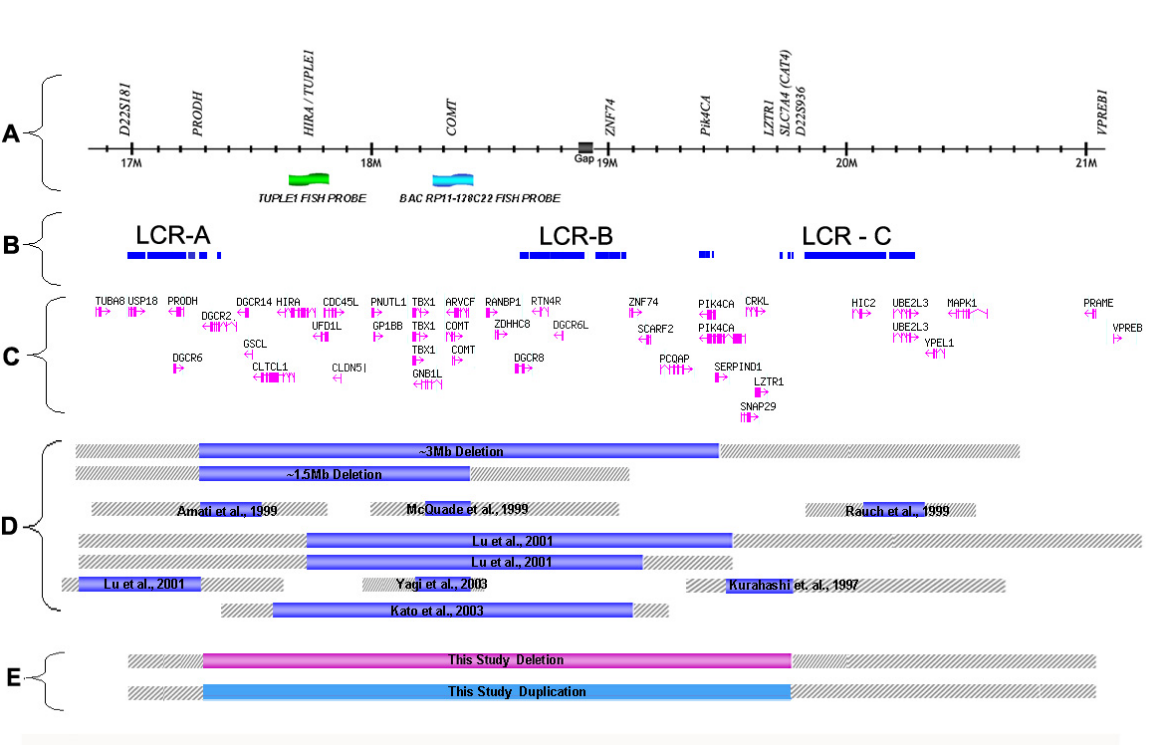
**Schematics of the 22q11.2 region**. Previously reported deletions and deletions identified from our study are shown. A) The 10 qPCR primers used to screen for hemizygous deletions, orientation is centromere to telomere. B) Known low copy number repeats or segmental duplications in 22q11.2: LCR-A, LCR-B and LCR-D (Shaikh et al., 2000). C) Known genes [24]. D) Location of previously reported deletions in 22q11DS patients. E) Locations of hemizygous deletions and duplications identified in this study. For D and E, hashed ends represent regions of uncertainty regarding precise location of deletion breakpoints.

The most critical factor for successful detection of micro-alterations using qPCR was primer design. To guarantee optimal primer design a high-resolution 22q11.2 physical map was constructed using information available from published reports [8-12,26-34] and online databases and repositories [16-24]. This allowed for the identification of unique sequences within the 22q11DS affected region whilst also avoiding the complex repetitive regions. Figure 1 shows a schematic representation of the 22q11.2 region studied in this work, the location of previously reported deletions and deletions identified from our study are shown as are the location of primers, repeat sequences, known genes and pseudogenes.

Sequences were selected to lie, mostly, within exonic and/or intervening regions from known or putative genes: UniSTS marker D22S181 (UniSTS is a comprehensive database of sequence tagged sites) [35], Proline Dehydrogenase/Proline Oxidase (PRODH), TUP-like enhancer of SPLIT 1 (TUPLE1), Catechol-O-Methyltransferase (COMT), Zinc Finger Protein 74 (ZNF74), Phosphatidylinositol 4-kinase (PIK4CA), Leucine-zipper-like Transcriptional Regulator 1 (LZTR1), Cationic Amino acid Transporter-4 (CAT4), D22S936 and Similar to Pre-B lymphocyte gene 1 (VPREB1). The selected sequences were aligned against the human genome using the BLAT program [36] to ensure that only contiguous sequences with 100% homology to one unique location were selected. A single primer set was then designed from each of these unique sequences using the Primer Express v 2.0 program (Applied Biosystems). We designed reference primers for each of the "housekeeping genes" Glucose-6-Phosphate Dehydrogenase (G6PDH) and Hydroxymethylbilane Synthase (HEM3) which were selected using published guidelines [37]; moreover we insured that they were single copy genomic sequences according to the BLAT alignment [38], in addition agarose gel visualization confirmed a single band of the expected size. All primer sequences are shown in Table 1. The use of reference primers was to control for varying input amounts of DNA from each separate patient. Thus any differences in the qPCR values obtained for test primers/markers would correspond to differences in the amount of the primers target sequence.

**Table 1:** Sequence and parameters of the reference and 22q11.2 test primer sets. Ten sets of primers were designed from within regions of unique sequence on 22q11.2 using Primer Express v2.0. In addition, two sets of reference primer for G6PDH and HEM3 were also designed to allow for data correction.

**Primer Sets**	**No.**	**Primer Name**	**Primer Sequence**	**Chr.**	**Genomic Location of Amplicon**	**Amplicon Size (bp)**	**Cycle Treshold**	**Slope**	**R2**
**Reference**	**1**	G6PDH-F	TCTTCATCACCACAGAGAACTTGC	1	9033513–9033733	221	0.2	-3.2968	0.9980
		G6PDH-R	GACCTGGAAGTCACTGGGCA						
	**2**	HEM3-F	TGCACGGCAGCTTAACGAT	11	118501618–118501818	202	0.2	-3.3998	0.9981
		HEM3-R	AGGCAAGGCAGTCATCAAGG						
**Test**	**1**	D22S181-F	CAGCTCCCAAGTCTTTCCAGC	22	16968759–16968859	101	0.2	-3.4141	0.9986
		D22S182-R	CCAGGGTAGGAAACAGGTCGA						
	**2**	PRODH-F	GGGAAAGGAGAGTTCAGGCAG	22	17293217–17293317	101	0.2	-3.5102	0.9992
		PRODH-R	GCTTGTTGAATAGCCTCTGTCCTAG						
	**3**	TUPLE1-F	GGCAAGTGCAATATTCATGTGGT	22	17763150–17763250	101	0.2	-3.2564	0.9996
		TUPLE1-R	TCCTACACGCCTGACAAAGCT						
	**4**	COMT-F	GTGCTACTGGCTGACAACGTGAT	22	18330640–18330739	100	0.2	-3.5876	0.9928
		COMT-R	GGAACGATTGGTAGTGTGTGCA						
	**5**	ZNF74-F	TGGCCTCCTGCTTCTTTCTTC	22	19073892–19073992	101	0.2	-3.2263	0.9960
		ZNF74-R	CAGACACTCCAATTCATGACGAA						
	**6**	PIK4CA-F	ATGCTTGTGCGACGCAGAC	22	19429805–19429905	101	0.06	-3.0238	0.9958
		PIK4CA-R	CCTCAGCCATGTTGACTCAGC						
	**7**	LZTR1-F	TCATCATGGATGTGTACAAACTGG	22	19673422–19673524	103	0.2	-3.2416	0.9977
		LZTR1-R	AGCACGTTCTGCAGGTCCAC						
	**8**	CAT4-F	TACCTGGGCTTCTTGGATGG	22	19708684–19708784	101	0.2	-3.4394	0.9986
		CAT4-R	AAGACAAGCACGCAGCCTATG						
	**9**	D22S936-F	TGGCAGCCAGTTTAGTATTCTGC	22	19777314–19777414	101	0.2	-3.1972	0.9960
		D22S936-R	TTGTAATCAAGTCCCGCCACT						
	**10**	VPREB1-F	CGACCATGACATCGGTGTGT	22	20924000–20924102	103	0.2	-3.2923	0.9972
		VPREB1-R	CTGGCTCTTGTCTGATTGTGAGA						

### Optimization process

Primer concentrations were optimised over a range of final concentrations, 100 nM to 900 nM at 100 nM intervals. The optimal concentration was that which obtained the lowest threshold cycle (C_t_) and maximum ΔR_n _while minimizing non-specific amplification. The results indicated that for all of the 22q11.2 primer sets the optimal final primer concentration was 800 nM, whilst for the G6PDH and HEM3 primer sets this value was 400 nM. The specificity of amplification for each qPCR product was confirmed by determining that the melting curve, showed a single dissociation peak corresponding to the melting temperature of the analysed amplicons (See Additional File 3, Figure 2 b. Dissociation Curve for PRODH -14 DNA samples).

To allow for comparisons between each primer sets all had to amplify with comparable efficiency. This was assessed by analysis of the slopes from the standard curves, which were generated using a log_10 _dilution series of input genomic DNA (range 10^2 ^nM to 10^-2 ^nM). If all conditions are optimal and reactions are 100% efficient, it will take approximately 3.32 cycles for ten fold amplification (log_2 _of 10 = 3.321928) of product, a value that is equal to the slope of the standard curve. This translates to 1 cycle to copy 1 molecule into 2; a second cycle to copy 2 molecules into 4; a third cycle to copy 4 into 8 and 0.32 cycles to copy 8 into 10. There was a linear relationship between the amount of input DNA and the threshold cycle (C_t_) values for the various reactions. Regression analyses of the C_t _values generated by the log_10 _dilution series gave R^2 ^values for all reactions in excess of 0.99 (Table 1).

At the optimal primer concentrations all of the primer sets gave slope values of 3.32 ± 0.25, indicating that the reactions were occurring with similar efficiencies. Following primer optimisation a baseline C_t _was identified for each primer set, which was used when analysing subsequent data (R^2^, slope and threshold cycle values are shown in Table 1).

### Data correction/normalization

To control for differences in sampling, DNA preparation, reaction efficiency (the varying PCR efficiencies between patient samples and the calibrator) [39] and other variables such as the C_t _values for each primer pair [40] from 22q11.2 were corrected/normalized using the C_t _value of the G6PDH and HEM3 products for the same sample. Although the input of template was standardised at 10 ng of DNA, the C_t _values for the housekeeping genes differed slightly from patient to patient and from group to group (Table 3), thus demonstrating the need for correction of the raw data prior to further analysis.

**Table 3:** Example of uncorrected Ct values characteristic to the reference (housekeeping) primer G6PDH. Exemplification of the uncorrected Ct (average of three replicates) values characteristic to the reference (housekeeping) primer G6PDH of the DNA samples under study. Although starting concentration of DNA for all samples was 10 ng the C_t _values for the housekeeping genes differed slightly from patient to patient and from group to group.

**Samples**		**G6PDH Ct value**
**Control**	**1**	21.2196
	**2**	21.1595
	**3**	23.0891
	**4**	21.3748

**Group 1**	**1**	19.8799
	**2**	21.2899
	**3**	20.5799
	**4**	20.7859
	**5**	21.4781
	**6**	19.5612

**Group 2**	**1**	20.3756
	**2**	21.1237
	**3**	21.1544
	**4**	21.1484
	**5**	20.5768
	**6**	19.9122

**22qDuplication**	**1**	21.3409

Correction was performed using a method described by Moody et al. (2000) [41]. Once the corrected C_t _values (KC_t_) for each of the test markers had been determined it was then possible to identify fold copy number change (ΔKC_t_) for each of the markers from the 22q11.2 region, using a formula described by Sijben et al. (2003) [42]. In the context of 22q11DS this approach calculates a ratio; by comparing the C_t _value obtained for each primer pair between the normal (control) DNA samples and the patient (affected) DNA samples. This value is then translated into fold changes (copy number gain or loss) per sample. For all calculations please refer to the Methods section.

### qPCR data

A summary of the qPCR results is presented in Table 2; light grey shading denotes loss, whilst dark grey shading denotes gain. We obtained ΔKC_t _values of either 0 ± 0.35 indicating an equal ratio of the target and reference, which corresponds to no loss and therefore no genetic abnormality, or -1 ± 0.35, indicating loss of one copy (microdeletion), for the affected samples. For the trisomy 22 patient we obtained a ratio of 1 ± 0.35, indicating gain of one copy. Each experiment was performed in triplicate, with replicates performed on different days. The inter-assay (same assay repeated on different days) C_t _variation and the intra-assay C_t _variation (the triplicates) was less than or equal to ± 0.35 cycles.

**Table 2:** Fold copy number change (ΔKC_t_) for the 12 22q11DS patients, 22q11 Duplication and 4 controls. ΔKC_t _values of 0 ± 0.35 indicate an equal ratio of the target and reference, which corresponds to no loss and therefore no genetic abnormality, values of -1 ± 0.35 indicate loss of one copy (microdeletion), whilst values of 1 ± 0.35 (seen for the trisomy 22 patient) indicate gain of one copy (microduplication).

**Sample Types**	**Samples**	**qPCR Primers of the 22q11.2 DS Region**									
		**D22S181**	**PRODH**	**TUPLE1**	**COMT**	**ZNF74**	**PIK4**	**LZTR1**	**CAT4**	**D22S936**	**VPREB1**
		Fold copy number change (ΔKCt)									
**Normal Controls**	**1**	-0.1601	0.2039	0.1716	0.1273	0.0744	0.1097	-0.1598	0.0175	-0.2195	0.1782
	**2**	0.0881	-0.0115	-0.0703	-0.0045	-0.0744	-0.1421	0.0538	-0.1002	-0.0327	0.1738
	**3**	-0.0802	-0.1322	-0.0202	0.0471	0.1633	0.0211	0.1497	-0.1193	0.1580	-0.1393
	**4**	-0.2727	-0.0602	-0.0812	-0.1700	0.2048	0.0113	-0.2035	-0.1173	-0.1508	-0.2128

**22q11DS Tuple1 Deleted**	**1**	-0.2006	-1.2337	-1.2173	-0.9261	-1.2366	-1.1903	-0.9009	-1.1467	-1.1305	0.0140
	**2**	0.1987	-1.1857	-1.2806	-1.1102	-0.8132	-0.9187	-1.2182	-0.8644	-1.1021	-0.0667
	**3**	-0.0035	-1.2003	-1.1671	-1.2660	-1.0857	-1.2183	-0.8808	-1.1124	-1.3388	-0.3315
	**4**	-0.0387	-1.2372	-1.2534	-1.2976	-0.9337	-1.1139	-1.1951	-0.9513	-0.9711	0.0759
	**5**	-0.2601	-0.9173	-1.1538	-0.9330	-0.9639	-0.9715	-1.2522	-1.0367	-1.1721	0.1191
	**6**	0.0158	-1.0541	-1.1710	-0.8799	-0.9787	-0.9763	-1.0172	-0.9620	-1.3164	0.1496

**22q11DS Tuple1 Not-Deleted**	**1**	0.0670	-0.1259	0.0717	-0.1508	0.2653	-0.0241	-0.1144	-0.1205	0.0881	0.0140
	**2**	-0.0325	-0.0797	-0.1033	-0.1055	0.1414	-0.0507	-0.1921	-0.1158	-0.0905	-0.0667
	**3**	-0.1568	-0.0003	0.1309	-0.1154	0.1521	-0.0615	-0.0400	0.1025	0.1660	-0.3315
	**4**	-0.0737	-0.1425	0.0046	0.0205	0.2222	-0.0246	-0.1283	0.1719	0.1093	0.0759
	**5**	0.1166	0.0283	-0.0596	0.1068	0.1951	-0.0478	-0.1887	0.1936	0.0282	0.1191
	**6**	-0.0508	0.1578	0.0755	0.0324	0.1366	-0.0066	0.0569	0.1882	0.0900	0.1496

**22q11 Duplication Sample**		-0.0124	1.1855	0.9201	0.9303	1.0121	0.9377	1.0648	0.9637	1.0250	-0.1863

## Discussion

In our analysis we have used a series of 12 qPCR primer sets to analyze twelve patients with clinical symptoms of 22q11DS and four controls. Ten of the primer sets (test primers) amplify markers localized within and around the chromosome 22q11.2-deleted region (D22S181, PRODH, TUPLE1, COMT, ZNF74, PIK4CA, LZTR1, CAT4, D22S936 and VPREB1) and two amplify "housekeeping" genes/markers (reference primers) (G6PDH and HEM3). The 22q11DS locus contains approximately 50 genes or pseudogenes and is characterized by an unusual genomic architecture comprising a large polymorphic chromosome-specific low copy repeats (Figure 1)[34]. The repetitive nature of this region of the genome is thought to increase the frequency of deletions and duplication events associated with clinical disease. The repetitive nature of the region of 22q11.2 under study required bioinformatics to identify unique regions for primer design.

In the context of 22q11DS we were trying to discriminate between 2 copies of a product (normal) versus 1 copy of the same product (deleted). In order to do this we had to implement data correction [41] to control for variations such as the input DNA concentration or reaction efficiencies [39]. To perform the correction we used the reference genes. The copy number of the reference genes will be the same in all of the samples under investigation. Any variation in copy number for the reference between samples will be the result of differences in initial template concentration, as long as the same DNA is sampled for both the control and target primers. Once the C_t _values for the reference were determined for each sample, it was then possible to use these to correct the values of the test markers for variation in initial template concentration. This correction permitted determination of copy number differences between the samples under investigation.

Individuals who had previously shown a deletion of TUPLE1 by FISH also showed deletion by qPCR (group 1). Our data demonstrates that the ΔKC_t _values, -1 ± 0.35, for the primers PRODH, TUPLE1, COMT, ZNF74, PIK4CA, LZTR1, CAT4 and D22S936 are indicative of deletion (Table 2). A finding that is in 100% concordance with the FISH results for the TUPLE1 probe. The region of deletion spanning PRODH, to D22S936, represents an interval of 2,502,410 base pairs (bp). Furthermore, the implementation of qPCR has allowed for the identification of breakpoint within the 22q11.2 region. The markers D22S181 and VPREB1 show values indicative of no deletion (0 ± 0.35), suggesting that the proximal deletion breakpoint occurs between the markers D22S181 and PRODH and the distal breakpoint between D22S936 and VPREB1 (see Table 2 and Figure 1).

The individuals that did not demonstrate loss using the TUPLE1 FISH probe were also deletion negative by qPCR, showing ΔKC_t _values similar to the normal controls (0 ± 0.35) thus indicative of no loss. For the 22q11 trisomy sample the qPCR results showed ΔKC_t _values indicative of duplication (1 ± 0.35) for PRODH, TUPLE1, COMT, ZNF74, PIK4CA, LZTR1, CAT4 and D22S936. The markers D22S181 and VPREB1 again showed values indicative of no copy number change (0 ± 0.35).

To accurately calculate fold changes, to the high precision required for genomic DNA quantitation, the real-time data analysis used here makes the most of the existing methods. The slope values determined from the standard curve allows for correction for variations in the primers efficiency and reaction kinetics, whilst the relative abundance ratio, calculated after the samples are normalized using the reference genes, allows for determination of gene/marker copy number. This rationale reduces, as much as possible, the unavoidable approximations introduced with any method of data interpretation.

The use of qPCR to detect and refine copy number differences in patients suffering from 22q11DS provides a further novel application for qPCR methodology. When used in a research setting, this type of analysis has proven to be very useful when comparing levels of a transcript or genomic markers [43,44] between different groups, and has been widely used in the study of human malignancies. However, such an approach has rarely been applied in a clinical setting to study constitutional deletions. The advantage of this technique over standard FISH assays is that qPCR provides a quantitative measurement of DNA copy number.

## Conclusion

Here we demonstrate the application of a robust, fast and accurate real-time quantitative PCR based assay using SYBR ^® ^Green I dye, that is capable of screening for copy-number alterations in genomic DNA.

Although qPCR detection methods have previously been used in 22q11.2 deletion analysis [45-47], these reports have only used a small number of primers/markers and have not been able to refine the region of deletion as has been done here. The utility of the approach outlined in this paper is the ease with which one can increase resolution by increasing the number of primers in the 22q11 deleted region thus facilitating accurate mapping of deletion breakpoints. The fine structure of qPCR mapping of deletions will reveal important clues into the mechanism by which the deletion occurs and thus will offer insights into the "at risk" factors predictive of deletions or other rearrangements.

The implementation of qPCR for genomic copy number profiling will provide a valuable tool for detection of atypical microdeletions and/or microduplications in individuals who go undiagnosed by the current available FISH methods. Such data will be useful for phenotype correlation studies. In addition, this methodology has the advantage of providing greater flexibility and adaptability than the currently available cytogenetic methods and will be beneficial in molecular classification and diagnosis.

## Methods

### Primer design

Primers (Table 1) were designed using Primer Express v2.0 (Applied Biosystems). The parameters for primer design were as follows; amplicons of 100–250 bp with a penalty score no higher than 10–12; primer melting temperature (*T*_m_) range 58°C to 60°C; primer length range 18 to 25 bp, optimal 20 bp; primer G/C content range 20% to 60%; amplicon maximum *T*_m _of 85°C. Runs of more than three identical nucleotides were avoided as polyG or polyC stretches can promote non-specific annealing, whilst runs of polyA and polyT can potentially open up stretches of the primer-template complex. Primers were required to be free of self-complementary sequence in order to avoid hairpin loop formation. In addition, no more than two G and/or C bases were permitted in the last five nucleotides at the 3' end to avoid GC clamp formation. Minimal deviation from the parameters was allowed only when there were no other options due to the complex nature of the 22q11.2 sequence. Primer and amplicon sequences were compared to the human genome using the BLAT program to guarantee that they showed 100% homology to only the sequence from which they were designed and also to guarantee that the forward and reverse primers were free of single nucleotide polymorphisms.

### Isolation of DNA

The DNA used in this study was obtained from the lymphocytes of four normal controls and 12 patients displaying clinical features of 22q11DS using phenol-chloroform extraction [48]. DNA was quality tested. Suitable sample for the qPCR reactions were of high molecular weight (un-sheared band of undigested DNA visible on a 0.5% agarose gel) and as clean as possible (an OD 260/280 ranging from 1.8 to 2.0).

### Reaction conditions

Reactions were performed using SYBR Green I PCR Master Mix (Applied Biosystems), which includes the internal reference (ROX). Each qPCR reaction comprised 12.5 μl 2× SYBR Green PCR Master Mix, forward and reverse primer at optimized concentrations of 800 nM (final concentration) for the 22q11.2 test primers and 400 nM (final concentration) for the reference primers, 10 ng/μl genomic DNA template and sterile water up to a final volume of 25 μl. The qPCR reactions were performed using the ABI Prism 7900 high-throughput sequence detection system. The reaction profile was: initial step, 50°C for 2 min, denaturation, 95°C for 10 min, then 40 cycles of denaturing at 95°C for 15 sec and combined annealing and extension at 60°C for 60 sec.

### Generating the standard curve

To generate standard curves for the selected primers and the reference primers a log_10 _dilution series of genomic DNA was prepared at concentrations ranging from 10^2 ^nM to 10^-2 ^nM. Each dilution was tested in triplicate. When analyzed by qPCR, the dilution series produced a set of standard curves, which were used to calculate the slope value with the aid of the SDS software version 2.1 Applied Biosystems (values are shown in Table 1). See Additional File 3, Figure 2 e. and f. for an example of SDS output report of standard and amplification plots.

### DNA quantification data analysis

Each qPCR experiment contained triplicates of the no-template-controls and patient samples for all of the primers tested. On the same reaction plate all DNA samples were tested with the test and reference primers. When any particular sample was being tested, the qPCR using reference primers and that sample were always included on the reaction plate. Each experiment was performed in triplicate, with replicates being performed on different days. Quantification was based on the increased fluorescence, which was measured and recorded using the ABI Prism 7900 sequence detection system and associated SDS software version 2.1 (Applied Biosystems). Results were expressed in terms of the threshold cycle value (C_t_; the cycle at which the change in fluorescence for the SYBR dye passes a significance threshold). The threshold values are shown in Table 1. The output of the results was exported in tab-delimited text file format. Further calculations were performed using Microsoft Excel. PCR products were resolved by agarose gel electrophoresis to confirm the presence of a single band of the expected size (See Additional File 3, Figure 2 a. showing SDS output of amplification plot for 14 DNA samples for the PRODH and G6PDH primer sets along with images of corresponding gel bands c. and d.).

### Data normalization

The qPCR data was normalized adapting a method devised by Moody et al. (2000) [41] and also described by Sijben et al. (2003) [42]. (See Additional File 1: Derivation of the formula)

KCti=(ACtR-CtRiSR)×ST+CtTi
 MathType@MTEF@5@5@+=feaafiart1ev1aaatCvAUfKttLearuWrP9MDH5MBPbIqV92AaeXatLxBI9gBaebbnrfifHhDYfgasaacH8akY=wiFfYdH8Gipec8Eeeu0xXdbba9frFj0=OqFfea0dXdd9vqai=hGuQ8kuc9pgc9s8qqaq=dirpe0xb9q8qiLsFr0=vr0=vr0dc8meaabaqaciGacaGaaeqabaqabeGadaaakeaacqWGlbWscqWGdbWqdaWgaaWcbaGaemiDaqNaemyAaKgabeaacqWGGaaikiabg2da9iabdccaGiabdccaGmaabmaabaWaaSqaaeaacqWGbbqqcqWGdbWqdaWgaaWcbaGaemiDaqNaemOuaifabeaakiabdccaGiabd2caTiabdccaGiabdoeadnaaBaaaleaacqWG0baDcqWGsbGucqWGPbqAaeqaaaGcbaGaem4uam1aaSbaaSqaaiabdkfasbqabaaaaaGccaGLOaGaayzkaaGaemiiaaIaey41aqRaemiiaaIaem4uam1aaSbaaSqaaiabdsfaubqabaGccqWGGaaicqGHRaWkcqWGGaaicqWGdbWqdaWgaaWcbaGaemiDaqNaemivaqLaemyAaKgabeaaaaa@53F7@

Where:

KC_ti _= 'Corrected *C*_*t*_' (*KC*_*t*_) of the test primer (*T*) against the reference (*R*)

AC_tR _= the Average C_t _value for Reference primer set for all the samples included in one qPCR run (control and patient).

C_tRi _= Ct value for Reference primer set for the sample to be corrected.

S_R _= slope value (from the standard curve) for reference primer set.

S_T _= slope value (from the standard curve) for test primer set.

C_tTi _= C_t _value for test primer set.

### Copy number calculation

Fold copy number (ΔKC_t_) change for each of the markers from the 22q11.2 region, was obtained using the formula:

Δ*KC*_*t *_= *KC*_*t*/*control *_- *KC*_*t*/*affected*_

ΔKC_t _= fold change (copy number gain or loss)

KC_t/control _= "Corrected C_t_" of the test primer for the control samples.

KC_t/affected _= "Corrected C_t_" of the test primer for the affected sample.

(Of note when multiple controls were used in the same reaction run the KC_t/control _was obtained by averaging all the controls'

"Corrected C_t_" – KC_t/control_'s). (See Additional File 2: Working example)

## Abbreviations

qPCR – Real time Quantitative Polymerase Chain Reaction

G6PDH – Glucose-6-Phosphate Dehydrogenase

Reference (R) – refers to the sequence we use to standardize against – the sequence, which we expect to remain unchanged in all the DNA samples (patient and control), and is the equivalent of the housekeeping gene use in expression studies. In our experiments we use G6PDH and HEM3.

Target (T) – refers to the sequence originating from the region of interest which we test and which we expect to find modifications (deletion or duplication).

## Authors' contributions

JS and RW conceived the study, and participated in its design, coordination and final writing; SH resourced the mathematical model and the methodology, revised the data and the manuscript for publication; LM developed the required bioinformatics, carried out the molecular genetics study, performed the data interpretation adapting the mathematical model, additional materials and, together with SH drafted the manuscript; ASB and EWCC contributed microdeletion cell lines, pertinent clinical information and contributed to overall study design; all authors revised and approved the final manuscript.

## Supplementary Material

Additional File 3 **Figure 2 (Fig. 2) Example of SDS output report and agarose gel for samples run on real-time qPCR**. a. Amplification plot for PRODH (14 DNA samples) and G6PDH (14 DNA samples). b. Dissociation Curve PRODH -14 DNA samples. c. and d. Agarose gel electrophoresis images showing unique bands for 14 DNA samples qPCR products corresponding to 101 bp fragment (PRODH primer set) and respectively 202 bp – G6PDH. e. COMT Standard Curve Plot and f. Amplification Plot for standard dilutions. Click here for file

Additional File 1 MS Word 2000 document demonstrating the mathematical derivation of the formula for copy number transition (fold change). Click here for file

Additional File 2 MS Word 2000 document describing the mathematical formula applied to a practical example (working example) where fold change is calculated from the Ct values of SDS output in excel. Click here for file
