# Supplementary figures and images for: A method for accurate detection of genomic microdeletions using real-time quantitative PCR
Source: BMC Genomics. 2005 Dec 13;6:180. doi: 10.1186/1471-2164-6-180 (PMC1327677; doi:10.1186/1471-2164-6-180)

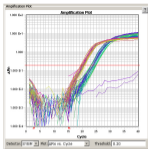

a)

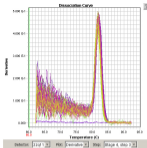

b)

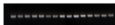

c)

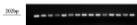

d)

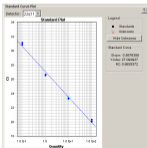

e)

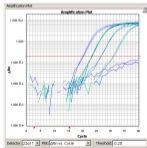

e)

Supplement: Additional File 3 — Figure 2 (Fig. 2) Example of SDS output report and agarose gel for samples run on real-time qPCR. a. Amplification plot for PRODH (14 DNA samples) and G6PDH (14 DNA samples). b. Dissociation Curve PRODH -14 DNA samples. c. and d. Agarose gel electrophoresis images showing unique bands for 14 DNA samples qPCR products corresponding to 101 bp fragment (PRODH primer set) and respectively 202 bp – G6PDH. e. COMT Standard Curve Plot and f. Amplification Plot for standard dilutions. [file 1471-2164-6-180-S3.pdf]
