# Supplementary material for: A method for accurate detection of genomic microdeletions using real-time quantitative PCR
Source: BMC Genomics. 2005 Dec 13;6:180. doi: 10.1186/1471-2164-6-180 (PMC1327677; doi:10.1186/1471-2164-6-180)
Supplement: Additional File 1 — MS Word 2000 document demonstrating the mathematical derivation of the formula for copy number transition (fold change). [file 1471-2164-6-180-S1.doc]

# Derivation of the formula

This part describes the mathematical derivation of the formula.

The fold changes per sample were calculated adapting the methods previously described (Moody et. al, 2000). The calculations and corrections were done similar as illustrated by Sijben and colabs. in 2003. However these methods had to be adapted to this paper specific application. The formula was derived with the purpose of providing a method for correcting for the sum of factors that adversely affect the real-time reaction efficiency – normalizing the recorded experimental C_t_ values.

If the reactions are 100% efficient, by converting the individual data using log_2_ ΔC_t_, this number will be ~3.32 cycles per log dilution, i.e.: log_2_ ΔC_t =_ log_2_ 10 =3.32192809488736 translated as: 1 cycle to copy 1 molecule into 2; a second cycle to copy 2 molecules into 4; a third cycle to copy 4 into 8 and 0.32 cycles to copy 8 into 10. However not all the primers will react with equal efficiency, so in order to correct for this (the equalizing factor) the following steps were taken in consideration:

***Normalizing the Reference:***

The following part of the equation describes how much individual samples tested for the reference or housekeeping gene deviate from the average; or the raw C_t_ difference observed in one particular individual compared to the average of all the individuals in one real-time PCR run:

or simplified

- represents the average C_t_ value for reference primer set of all samples (patient plus control) in one real-time PCR run;

*N* is the total number of DNA samples in one real-time PCR run (i.e. if we run DNA samples from 14 individuals in one experiment *N*=14).

*C_tRi_* = *C_t_* (cycle threshold) value for the reference primer set

Next we factor in the experimental variation reflected in the reference standard curve (*S_R_*) value adjusting the specific *C_t_* value (counting how many cycles it takes to generate a log 10 of DNA).

The equation becomes:

and it represents how much each DNA sample tested with reference primer set deviates from the threshold.

S_R_ is the slope value for the reference.

***Normalizing the target in rapport to the control***

To calculate the real value of how much each sample tested with the test primer deviate from the threshold (how many more or less cycles the sample need to amplify enough DNA to behave with the same efficiency) the equation will take in consideration the slope value of the test primer. Hence

represents the deviation from the point at which the test primers target fluorescence reaches the threshold - the theoretical point at which all the test primers perform with comparable efficiency.

S_T_ -represents the slope value for the test primer.

Finally the equation that describes the ‘Corrected *C_t_*’ (*KC_t_*) of the test primer (*T*) against the reference (*R*) is obtained by the sum of the experimental or recorded *C_t_* and the above-described deviation:

and represents the real value (normalized) of the C_t_, the crossing point of fluorescence at which *theoretically all the samples behave with similar efficiency*.

*C_tTi_* = the experimental C_t_ value for the target.

## Copy number transition calculation (fold change)

Finally we compare the target (patient sample) and control (normal controls) results to obtain the changes in the patient samples as compared to the normal DNA (fold change).

*KC_t / control_* = “Corrected C_t_” of the test primer for the control samples.

*KC_t / affected_* = “Corrected C_t_” of the test primer for the affected sample.

Where *ΔKC_t_* – represents copy number gain or loss per sample (fold changes).

In conclusion *ΔKC_t_* values of 0, (± 0.35) indicating an equal ratio of the test and reference, which corresponds to no loss and therefore no genetic abnormality, or -1 (± 0.35), indicating loss of one copy (microdeletion), for the affected samples. Similarly a ΔKC_t_ values of +1, (± 0.35) would indicate copy gain consistent with microduplication.

Note: In order to test for a better correlation multiple controls can be used on the same run (i.e. 4). The corrected *KC_t / control_* final values for each of them should be very close (± ~0.35), however for the final calculation these are averaged; the average of the control’s “Corrected C_t_” is than used for final fold change calculation.

Please refer to the “Working Example” for a calculation example.

**Definitions:**

**Control:** normal DNA or normal control;

**Target:** patient DNA;

**Reference primers or reference:** also dubbed housekeeping primers representing the primers that amplify loci that contain no change (in the context of this paper as all the experiments are done on genomic DNA it means that there are no deletions or other events such as duplications that change the sequence length);

**Test primers:** DNA sequences that amplify loci that may have undergone a change at genomic level (deletions, duplications) primers selected from the 22q11.2 region in the example of this paper;
