# Supplementary material for: A method for accurate detection of genomic microdeletions using real-time quantitative PCR
Source: BMC Genomics. 2005 Dec 13;6:180. doi: 10.1186/1471-2164-6-180 (PMC1327677; doi:10.1186/1471-2164-6-180)
Supplement: Additional File 2 — MS Word 2000 document describing the mathematical formula applied to a practical example (working example) where fold change is calculated from the Ct values of SDS output in excel. [file 1471-2164-6-180-S2.doc]

# File 2

# Working example- calculation

|  |  | **A** | **B** | **C** | **D** | **E** | **F** | **G** | **H** |
| --- | --- | --- | --- | --- | --- | --- | --- | --- | --- |
|  | **N** | Samples | C_tR_ - G6PDH |  | C_tT_ - TUPLE1 |  | KC_t_ |  | ΔKC_t_ |
|  |  | C1 | 21.909996 |  | 25.710926 |  |  |  |  |
|  | **1** | C1 | 21.936258 | 21.93083733 | 25.715622 | 25.741841 | 23.68289011 |  | -0.00549989 |
|  |  | C1 | 21.946258 |  | 25.798975 |  |  |  |  |
| Controls |  | C3 | 20.442316 |  | 24.17797 |  |  |  |  |
|  | **2** | C3 | 20.5943 | 20.47956233 | 24.046497 | 24.1096367 | 23.59459139 | 23.6773902 | 0.082798828 |
|  |  | C3 | 20.402071 |  | 24.104443 |  |  |  |  |
|  |  | C4 | 19.997199 |  | 23.749699 |  |  |  |  |
|  | **3** | C4 | 19.919315 | 19.973605 | 23.697824 | 23.7314833 | 23.75468916 |  | -0.07729894 |
|  |  | C4 | 20.004301 |  | 23.746927 |  |  |  |  |
|  |  | TDel1 | 18.812428 |  | 23.494623 |  |  |  |  |
|  | **4** | TDel1 | 18.75386 | 18.80831333 | 23.510918 | 23.516744 | 24.77961864 |  | **-1.10222842** |
|  |  | TDel1 | 18.858652 |  | 23.544691 |  |  |  |  |
|  |  | TDup | 20.492771 |  | 23.24345 |  |  |  |  |
|  | **5** | TDup | 20.48895 | 20.45689033 | 23.202683 | 23.248206 | 22.75727981 |  | **0.920110408** |
|  |  | TDup | 20.38895 |  | 23.298485 |  |  |  |  |
|  |  | TND1 | 20.388985 |  | 23.920969 |  |  |  |  |
|  | **6** | TND1 | 20.407995 | 20.37010667 | 24.09124 | 24.004254 | 23.60565062 |  | 0.071739595 |
|  |  | TND1 | 20.31334 |  | 24.000553 |  |  |  |  |
|  |  | TND2 | 20.423384 |  | 24.340689 |  |  |  |  |
|  | **7** | TND2 | 20.380028 | 20.42328167 | 24.1353 | 24.2358847 | 23.78071229 |  | -0.10332207 |
|  |  | TND2 | 20.466433 |  | 24.231665 |  |  |  |  |
|  |  | oDNA1 | 18.503317 |  | 22.287395 |  |  |  |  |
|  | **8** | oDNA1 | 18.55378 | 18.529149 | 22.338343 | 22.3362597 | 23.89611688 |  | -0.21872666 |
|  |  | oDNA1 | 18.53035 |  | 22.383041 |  |  |  |  |
|  |  | TND3 | 20.32131 |  | 23.90469 |  |  |  |  |
| Affected | **9** | TND3 | 20.424517 | 20.36914833 | 23.968155 | 23.944065 | 23.54648113 |  | 0.130909094 |
|  |  | TND3 | 20.361618 |  | 23.95935 |  |  |  |  |
|  |  | oDNA2 | 19.979033 |  | 23.527931 |  |  |  |  |
|  | **10** | oDNA2 | 19.89502 | 19.94261533 | 23.53838 | 23.5209973 | 23.57717081 |  | 0.100219413 |
|  |  | oDNA2 | 19.953793 |  | 23.496681 |  |  |  |  |
|  |  | oDNA3 | 19.508224 |  | 23.02334 |  |  |  |  |
|  | **11** | oDNA3 | 19.495731 | 19.53693933 | 23.695251 | 23.242511 | 23.73025358 |  | -0.05286336 |
|  |  | oDNA3 | 19.606863 |  | 23.008942 |  |  |  |  |
|  |  | TND4 | 19.723406 |  | 23.342966 |  |  |  |  |
|  | **12** | TND4 | 19.528376 | 19.603404 | 23.266027 | 23.2557207 | 23.67275634 |  | 0.004633883 |
|  |  | TND4 | 19.55843 |  | 23.158169 |  |  |  |  |
|  |  | TND5 | 19.234512 |  | 23.170435 |  |  |  |  |
|  | **13** | TND5 | 19.10723 | 19.36106 | 23.076271 | 23.0621803 | 23.73702811 |  | -0.05963789 |
|  |  | TND5 | 19.741438 |  | 22.939835 |  |  |  |  |
|  |  | TND6 | 20.454271 |  | 24.115068 |  |  |  |  |
|  | **14** | TND6 | 20.415268 | 20.425233 | 24.080267 | 24.0591717 | 23.60192341 |  | 0.075466813 |
|  |  | TND6 | 20.40616 |  | 23.98218 |  |  |  |  |
|  |  | oDNA4 | 19.566044 |  | 23.469355 |  |  |  |  |
|  | **15** | oDNA4 | 19.723646 | 19.72113233 | 23.307606 | 23.39743 | 23.68922308 |  | -0.01183286 |
|  |  | oDNA4 | 19.873707 |  | 23.415329 |  |  |  |  |
|  |  |  | || |  |  |  |  |  |  |
|  |  | ACtR | 19.9954185 |  |  |  |  |  |  |

Definitions, notations and explanations:

All samples were run in triplicates. Column B and D contain Ct values for ‘reference’ and ‘test’ from the qPCR SDS output in excel; C and E average of triplicates.

C 1 to 4 - 3 controls

TDel = Tuple1 FISH Deleted samples for 22q11

TDup= chromosome 22q duplication sample

TND = Tuple1 FISH Non-deleted samples for 22q11

oDNA = other sample runs not included in this paper

S_R_= 3.0609932 Slope value for the reference primer (G6PDH) – inferred from the G6PDH standard curve

S_T_= 3.2563674 Slope value for the test primer (TUPLE1) – inferred from the TUPLE1 standard curve

N=15 – total number of DNA samples in present run

Example of calculation for sample TDup where i=5 :

***‘Corrected C_t_’ (KC_t_) of the test primer (T) against the reference (R):***

= $\left(\frac{\text{19.9954185-20.45689033}}{\text{3.0609932 }}\right)\times3.2563674+23.248206=\text{22.75727981 }$

## Copy number transition calculation (fold change) of test sample compared to the control(s):

KC_t/contro l_= 23.6773902 – average of all

- value consistent with 3 copies (see column E)
